# Dietary emulsifier consumption alters gene expression in the amygdala and paraventricular nucleus of the hypothalamus in mice

**DOI:** 10.1038/s41598-022-13021-7

**Published:** 2022-06-01

**Authors:** Amanda R. Arnold, Benoit Chassaing, Bradley D. Pearce, Kim L. Huhman

**Affiliations:** 1 Neuroscience Institute, Georgia State University, Atlanta, GA 30303 USA; 2 INSERM U1016, Team “Mucosal Microbiota in Chronic Inflammatory Diseases”, CNRS UMR 8104, Université de Paris, Paris, France; 3 Department of Epidemiology, Emory University Rollins School of Public Health, 1518 Clifton Rd NE, Atlanta, GA 30322 USA

**Keywords:** Molecular neuroscience, Neuroimmunology

## Abstract

Dietary emulsifier consumption promotes systemic low-grade inflammation, metabolic deregulation, and possibly an anxiety-like phenotype. The latter finding suggests that dietary emulsifiers impact brain areas that modulate stress responses. The goal of the current study was to test whether emulsifier consumption is associated with changes in gene expression in the amygdala and the paraventricular nucleus of the hypothalamus (PVN), two brain areas that are involved in behavioral and neuroendocrine responses to stress. Using RNA-Seq, we compared groups consuming either carboxymethylcellulose or polysorbate 80 for 12-weeks. A total of 243 genes were differentially expressed in the amygdala and PVN of emulsifier-treated mice compared to controls. There was minimal overlap of differentially expressed genes in CMC- and P80-treated animals, suggesting that each emulsifier acts via distinct molecular mechanisms to produce an anxiety-like phenotype. Furthermore, gene ontology and pathway analysis revealed that various stress, metabolic, and immune terms and pathways were altered by emulsifiers. These findings are the first to demonstrate that emulsifier consumption changes gene expression in brain regions that are critical for stress responding, providing possible molecular mechanisms that may underly the previously observed anxiety-like phenotype.

## Introduction

Chronic low-grade inflammation has recently been identified as a key contributing factor in the etiology and progression of neuropsychiatric disorders^1,2^. The immune system interacts with stress regulatory brain areas to affect brain and behavior by altering neurochemical signaling, neuroplasticity, and neuroendocrine processes^3,4^. One common source of low-grade inflammation originates from the gastrointestinal tract when the host/microbiota interaction is altered through agents such as dietary factors. Indeed, select dietary components can lead to chronic, low-grade inflammation and may thereby increase susceptibility to developing a neuropsychiatric disorder^2,5^. Food and drink additives, such as emulsifiers, are commonly added to processed foods and many beverages to improve texture, consistency, and to extend shelf life. Although these additives are generally viewed as innocuous and are classified by the Food and Drug Administration as Generally Recognized As Safe, it has been recently reported that these additives at translationally relevant doses can cause systemic inflammation^5–7^. Specifically, consumption of emulsifiers, such as carboxymethylcellulose (CMC) and polysorbate-80 (P80), appears to reduce microbial diversity and to increase inflammation-promoting proteobacteria. CMC and P80 appear to act through different mechanisms on the intestinal microbiota in a way that promotes chronic intestinal inflammation that manifests as colitis in genetically susceptible mice and metabolic deregulations in mice that are not genetically susceptible^6,8^. Importantly, these phenotypes are abolished in germ-free mice and are transferrable via microbiota transplant from emulsifier-treated mice to germ-free mice, suggesting that the intestinal microbiota is both necessary and sufficient to drive emulsifier-induced detrimental effects^6,8^. Interestingly, previous data suggest that emulsifier-treated animals may also display a baseline anxiety-like behavioral phenotype^7^, suggesting that emulsifier-induced shifts in the microbiome may also impact the brain. The mechanism whereby emulsifiers contribute to the development of an anxiety-like behavioral phenotype, however, has yet to be determined.

Stress-related disorders are often characterized by dysregulation of the hypothalamic-pituitary-adrenocortical (HPA) axis^9^, the neuroendocrine cascade that is a key modulator of the body’s response to stress^9^. Furthermore, the HPA axis has a bi-directional relationship with the innate immune system as well as with the gut microbiota^10,11^. Sustained activation of the HPA axis can affect gut microbial composition and gastrointestinal permeability^12^. Conversely, antibiotic-induced gut dysbiosis can sensitize the HPA axis, resulting in excessive stress hormone release after acute stress exposure^13^. It is possible that altered gene expression in areas of the brain that play a major role in modulating anxiety and stress responding, such as the amygdala and paraventricular nucleus of the hypothalamus (PVN), may be responsible for the anxiety-like behavioral phenotype following ingestion of emulsifiers. It is also possible that emulsifiers induce inflammation in the brain and that this contributes to an altered behavioral and neuroendocrine stress response, as many studies have demonstrated that alterations in neuro-immune signaling pathways can affect stress responding^14,15^. Hence, the purpose of this present study was to test whether consumption of emulsifiers at doses used by the food industry are sufficient to alter gene expression in the brain in a way that might illuminate how these compounds alter stress responding and anxiety-like states. Using RNA-sequencing, we investigated whether emulsifier consumption alters gene expression in two critical stress-modulatory brain regions, the amygdala and PVN. We hypothesized that ingestion of emulsifiers alters immune and stress-related gene expression in both the amygdala and PVN. Overall, we present a comprehensive, genome-wide analysis of differentially expressed genes after emulsifier consumption in two brain regions that modulate the stress response.

## Results

### Verification of emulsifier-induced metabolic syndrome and low-grade inflammation

**Figure 1:**
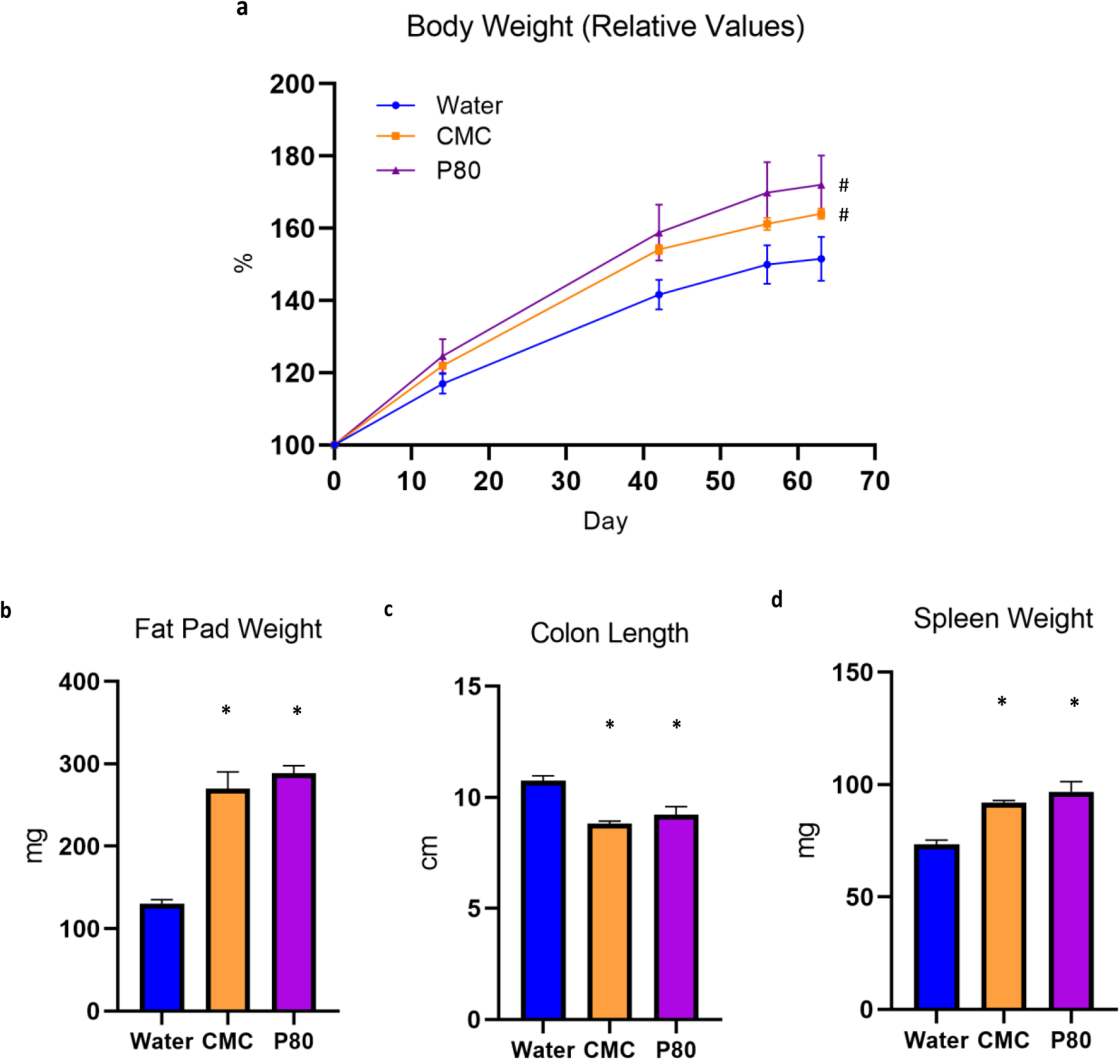
Emulsifier consumption results in metabolic syndrome and low-grade inflammation. (**a**) Relative body weight over time. Body weight is expressed as a percentage compared to the initial body weight (Day 0) defined as 100%. Ingestion of emulsifiers significantly increased fat-pad mass (**b**), decreased colon length (**c**), and increased spleen weight (**d**) compared to controls. Significance was determined using a repeated measure one-way ANOVA with a Geisser-Greenhouse correction (^#^p < 0.05) or unpaired t-tests (*p < 0.05). N = 5 in each group.

To verify that the animals used in the RNA-Seq and qRT-PCR validation experiment display the previously described low-grade inflammation and altered metabolic phenotype, the body weight of each subject was measured throughout the duration of the study and organs were weighed at the time of sacrifice. Animals that consumed emulsifiers showed a significant increase in weight over time (Fig. 1a; p = 0.0279) and increased adiposity, measured by fat pad weight (Fig. 1b; CMC vs Water, p = 0.0002 and P80 vs Water, p = < 0.0001). Mice that consumed emulsifiers also had significantly shorter colons (Fig. 1c; CMC vs Water, p = < 0.0001 and P80 vs Water, p = 0.008) and increased spleen weight (Fig. 1d; CMC vs Water, p = < 0.0001 and P80 vs Water, p = 0.0015), a phenotype that results from low-grade inflammation. Altogether, these data verify that the animals used in the RNA-Seq and qRT-PCR validation study display a phenotype consistent with metabolic syndrome and low-grade inflammation.

### Consumption of dietary emulsifiers alter hypothalamic and amygdala gene expression

**Figure 2:**
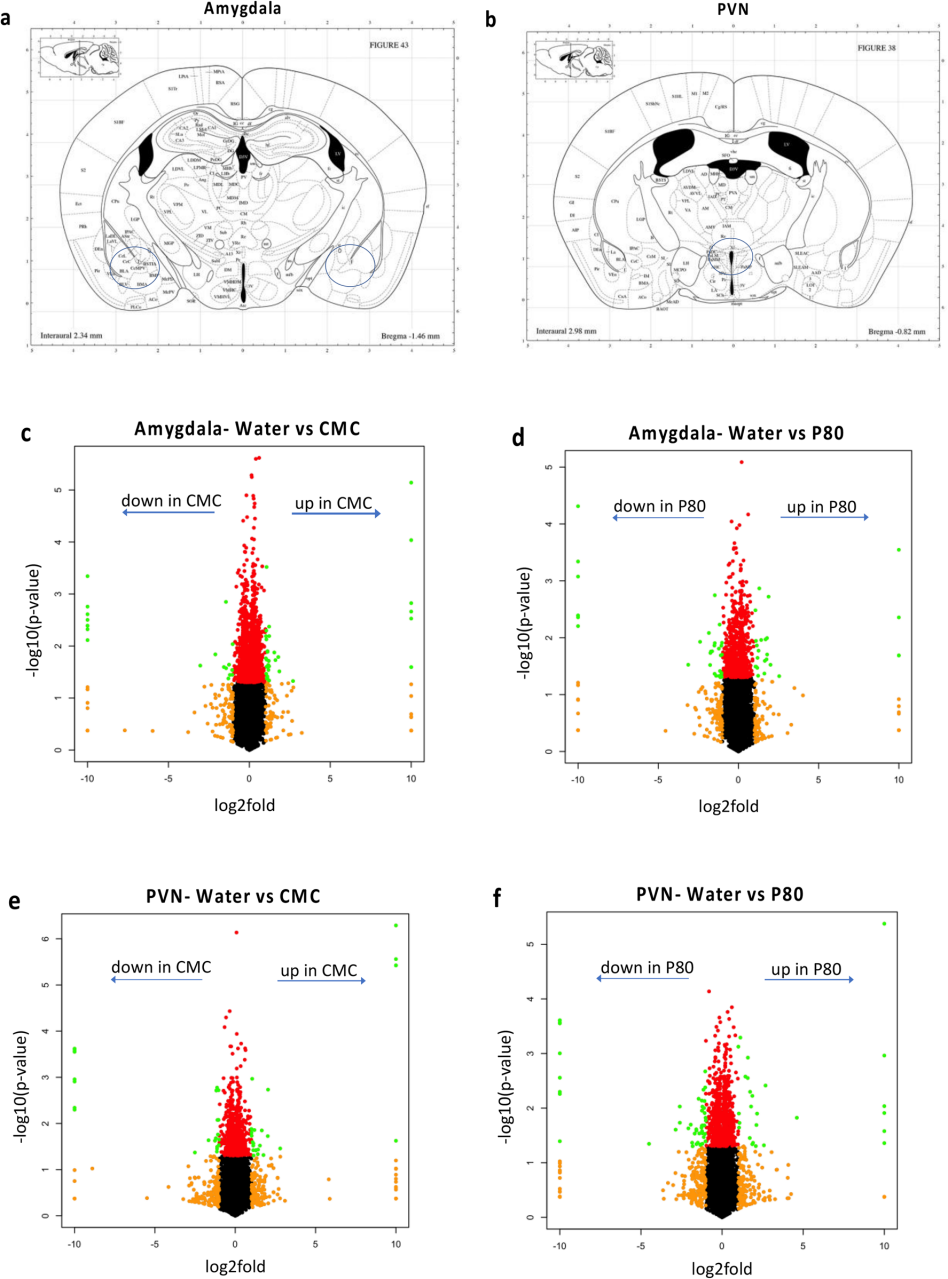
Dietary emulsifier consumption alters gene expression in the amygdala and paraventricular nuclei of the hypothalamus. Wild-type (WT) mice were exposed to plain drinking water or water containing CMC or P80 (1.0%) for 12 weeks and brain tissue was harvested. The extent of tissue punch in the amygdala (**a**) and PVN (**b**). Sections adapted from Paxinos, George, and Franklin 2001. Total RNAs were extracted, mRNAs purified, and subjected to library preparation and sequencing. Genes were filtered to keep only genes expressed in at least one condition (average FPKM (Fragments Per Kilobase Million) > 1 in at least one group) and were visualized on volcano plots. (**c**) Water-treated versus CMC-treated, amygdala. (**d**) Water-treated versus P80-treated, amygdala. (**e**) Water-treated versus CMC-treated, paraventricular nucleus. (**f**) Water-treated versus P80-treated, paraventricular nucleus. For each gene, the difference in abundance between the two groups is indicated in log2 fold change on the x-axis (with positive values corresponding to an increase in the emulsifier-treated group compared with the water-treated group, and negative values corresponding to a decrease in the emulsifier-treated group compared with the water-treated group). Log2 fold values that exceeded 10 or -10 are recorded as 10 or − 10. Significance between the two groups is indicated by − log10 p-value on the y-axis. Red dots correspond to genes with adj. p < 0.05 between emulsifier-treated and water-treated groups. Orange dots correspond to genes with at least a onefold decreased or increased expression in the emulsifier-treated group compared with the water-treated group. Green dots represent differentially expressed genes (adj. p < 0.05, log2 FC > 1 and < − 1) between control and emulsifier groups. Only genes in green were used for subsequent analysis.

**Figure 3:**
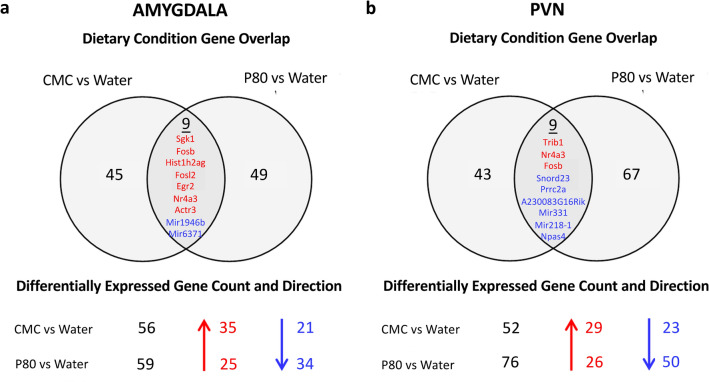
Quantified DEGs in the amygdala and PVN after treatment with P80 or CMC. Quantified genes were differentially expressed and met analysis criteria with an adjusted p-value of less than 0.05 and exceeded log2fold change of 1 and − 1. Venn-diagram shows the overlap of significant differentially expressed genes between CMC and P80 treatments. Upregulated DEGs are in red and downregulated DEGs are in blue.

**Figure 4:**
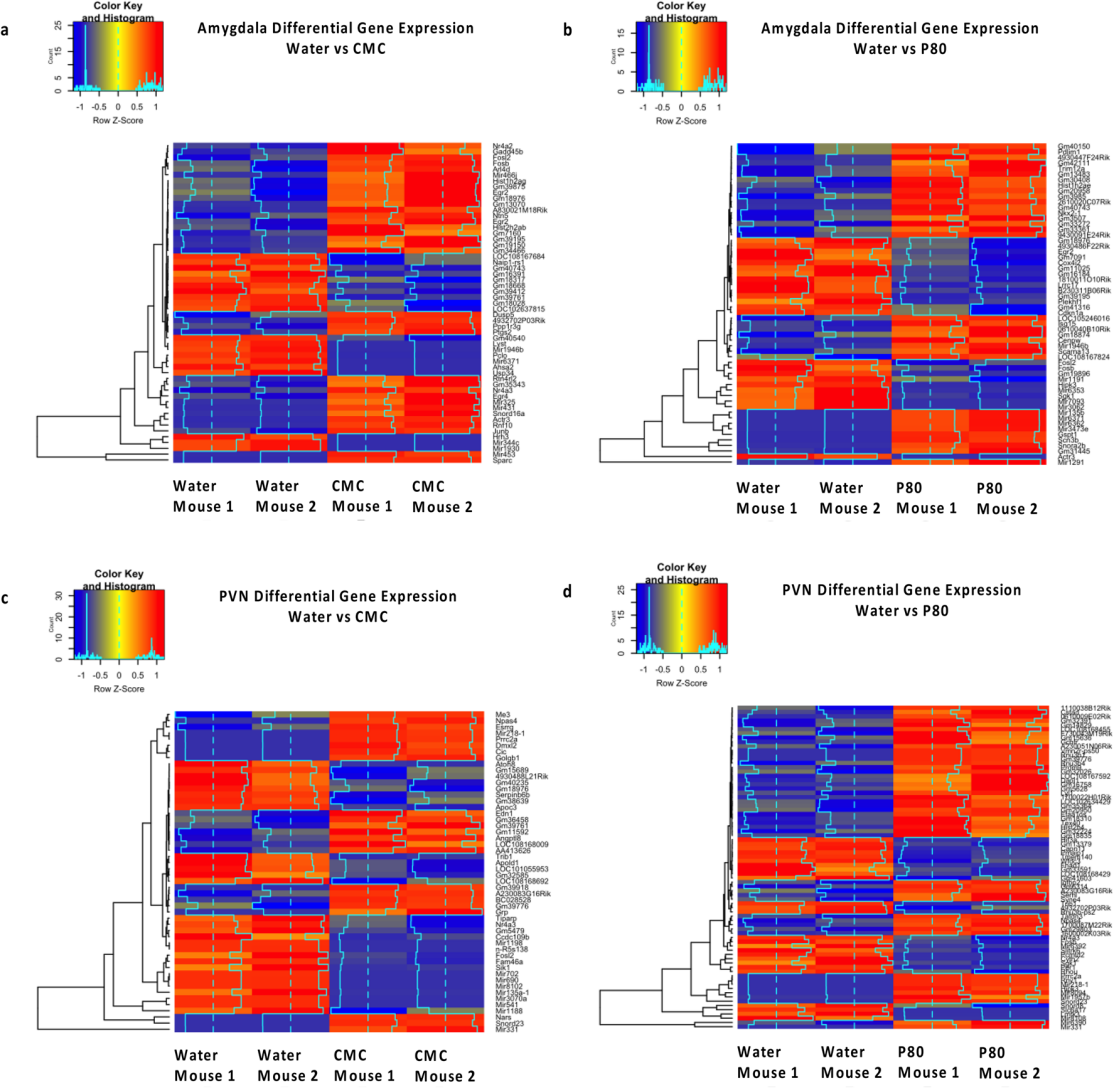
Heatmaps show z-score normalized relative expression of differentially expressed genes that met criteria (FC > 1, adj.p-value < 0.05). Hierarchical clustering based on Euclidian distance of 56 differentially expressed genes in the amygdala after CMC treatment and 59 with P80 treatment. 52 differentially expressed genes in the PVN after CMC treatment and 76 with P80 treatment are listed. Genes are indicated in rows and animals are in columns. Blue indicates downregulation and red upregulation of the differentially expressed genes. Heatmaps were generated using the gplots and RColorBrewer libraries in R.

**Figure 5:**
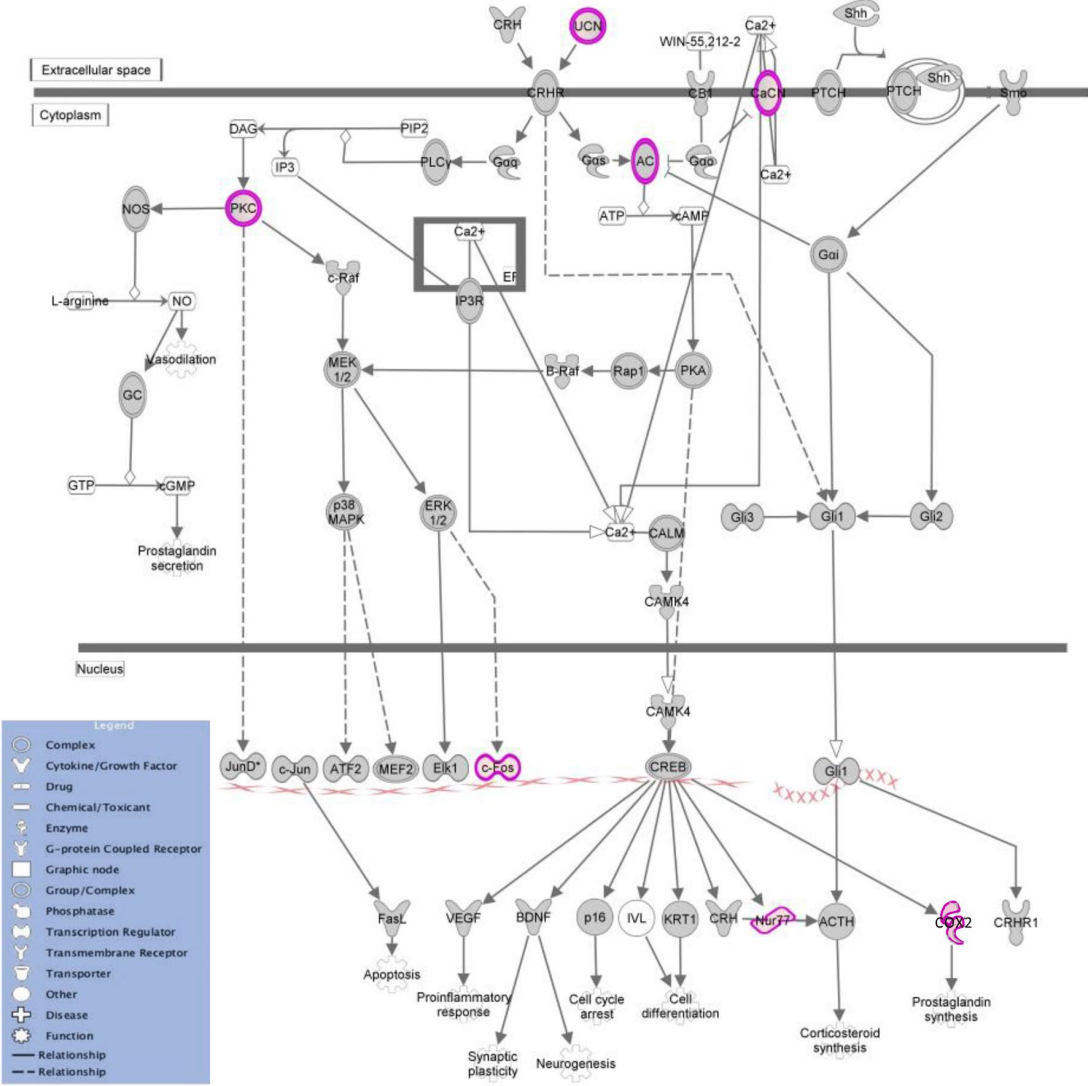
Corticotropin-releasing hormone pathway identified by Ingenuity Pathway Analysis. The CRH pathway is identified as one of the significant pathways by IPA within the PVN of CMC-treated animals (p = 9.13E−05). All up-regulated DEGs in our dataset are shown here in pink and overlaid onto the CRH molecular pathway.

To identify potential mechanisms by which emulsifier exposure could alter anxiety-like behavior, we applied an unbiased approach and performed total mRNA sequencing of the amygdala and PVN regions, known to play a central role in stress-responding and anxiety-like behaviors. Importantly, we observed that emulsifier consumption is associated with the modulation of gene expression in the amygdala and the PVN. Volcano plots and Venn diagrams were generated to broadly show the number of genes that were changed in the amygdala and PVN after each emulsifier treatment compared to water-only controls. Figure 2 demonstrates that a relatively low number of genes were found to be significantly differentially expressed (padj < 0.05, Log2 FC > 1 and < − 1) in the amygdala (Fig. 2a,c,d) and PVN (Fig. 2b,e,f), with the first two panels showing the anatomic localization of the tissue punches. Only genes that fit differential expressed gene (DEG) criteria were used for subsequent analyses (Figs. 3, 4, 5 and Tables 1, 2, 3). As presented in Fig. 3, the number of shared and different DEGs between emulsifier conditions in each brain region is relatively low compared to the total number of variables studied (18,778). In total, 243 genes were differentially expressed in the amygdala and PVN of emulsifier-treated animals compared to control animals. Counts include 56 unique DEGs in the amygdala after CMC treatment and 59 with P80 treatment. In addition, 52 unique DEGs were counted in the PVN after CMC treatment and 76 with P80 treatment. 9 DEGs were shared between CMC- and P80-treated mice in the amygdala and 9 DEGs were shared between CMC- and P80-treated mice in the PVN. Importantly, of the differentially expressed genes shared in common between CMC and P80, several were immediate early genes (IEGs), which are widely used as a molecular marker of neuronal activity. Emulsifier treatment increased expression of IEGs such as NR4A3, EGR2, FOSB, and FOSL2 in the amygdala and PVN, suggesting that both CMC and P80 may increase neuronal activity in these brain regions. A hierarchical heat map (Fig. 4), grouping genes with similar expression profiles into clusters, was included to show the direction of differential expression of each DEG and global DEG patterns occurring within (comparing individuals within each group) and between (water versus emulsifier) conditions.

### Gene ontology

To explore the potential biological meaning of the identified differentially expressed genes, we next used EnrichR to perform gene ontology to identify biological processes or molecular functions (Tables 1 and 2) involved^16–18^. Analysis of gene ontology yielded statistically significant (p-value < 0.05) enriched terms. An important stress and immune function term, “glucocorticoid receptor binding” was enriched in both CMC and P80 conditions, which may suggest possible HPA-axis dysfunction under both emulsifier conditions. DEGs known to code for protein that regulate glucocorticoid receptor (GR) binding include Nuclear Receptor Subfamily 4 Group A Member 3 (NR4A3, ^), Nuclear Receptor Subfamily 4 Group A Member 2 (NR4A2, ^), and Serum- and Glucocorticoid-inducible Kinase 1 (SGK1, ^). Specifically, NR4A3 and NR4A2 transcription factors inhibit GR-dependent repression of the POMC gene, a gene that encodes the precursor protein needed to synthesize ACTH. Further, both NR4A3 and NR4A2 have been shown to regulate HPA-axis neuroendocrine activity within the PVN, pituitary, and adrenals^19^. SGK1 potentiates and prolongs GR activation following cortisol stimulation by increasing GR phosphorylation and nuclear translocation^20^. SGK1 is both a downstream target of GR signaling and also affects GR activation by positively regulating the long-lasting effects of glucocorticoids^21^. The increase in differential expression of the above-mentioned genes in our dataset may suggest alterations in HPA-axis activity in animals that consume emulsifiers. Interestingly, several metabolism-related pathways were also enriched in the PVN as a result of emulsifier consumption. For example, for the CMC-induced deregulated DEGs, these pathways included “regulation of triglyceride metabolic process,” “regulation of lipoprotein lipase activity,” “regulation of lipid biosynthetic process,” “acylglycerol homeostasis,” and “triglyceride homeostasis,” whereas the P80-induced deregulated DEGs included metabolic-related terms such as “insulin-like growth factor” I and II binding. These data suggest possible neural mechanisms that may contribute to the dysfunctional metabolic phenotype previously described in emulsifier-treated mice^6^.

**Table 1:** Gene ontology of differentially expressed genes in the amygdala of emulsifier treated mice.

Gene ontology functional enrichment analysis			
Amygdala—CMC vs water			
GO ID	Biological process description	p-value	DEGs
GO:0031643	Positive regulation of myelination	4.02E−04	**RNF10;EGR2**
GO:0045444	Fat cell differentiation	5.46E−04	**NR4A2;EGR2;NR4A3**
GO:0031646	Positive regulation of nervous system process	1.10E−03	**RNF10;EGR2**
GO:0031641	Regulation of myelination	2.31E−03	**RNF10;EGR2**
GO:0099643	Signal release from synapse	6.24E−03	*PCLO;HRH3*
GO:0007269	Neurotransmitter secretion	6.52E−03	*PCLO;HRH3*
GO:0031620	Regulation of fever generation	1.37E−02	**PTGS2**
GO:0031394	Positive regulation of prostaglandin biosynthetic process	1.37E−02	**PTGS2**
GO:1903208	Negative regulation of hydrogen peroxide-induced neuron death	1.37E−02	**NR4A3**
GO:2000253	Positive regulation of feeding behavior	1.37E−02	**NR4A3**

GO ID	Molecular function description	p-value	DEGs
GO:0035259	Glucocorticoid receptor binding	2.64E−04	**NR4A2;NR4A3**
GO:0001228	DNA-binding transcription activator activity, RNA polymerase II-specific	2.19E−03	**NR4A2;EGR2;NR4A3;EGR4;FOSL2**
GO:1990837	Sequence-specific double-stranded DNA binding	1.31E−02	**NR4A2;RNF10;EGR2;EGR4;FOSB;JUNB**
GO:0016907	G protein-coupled acetylcholine receptor activity	1.91E−02	*HRH3*
GO:0099528	G protein-coupled neurotransmitter receptor activity	1.91E−02	*HRH3*
GO:2001069	Glycogen binding	2.18E−02	**PPP1R3G**
GO:0017017	MAP kinase tyrosine/serine/threonine phosphatase activity	2.72E−02	**DUSP5**
GO:0008330	Protein tyrosine/threonine phosphatase activity	2.72E−02	**DUSP5**
GO:0035497	cAMP response element binding	2.72E−02	**NR4A3**
GO:0061665	SUMO ligase activity	2.72E−02	**EGR2**

Amygdala—P80 vs water			
GO ID	Biological process description	p-value	DEGs
GO:0048539	Bone marrow development	1.49E−02	**LRRC17**
GO:0086018	SA node cell to atrial cardiac muscle cell signaling	1.49E−02	*SCN3B*
GO:0014037	Schwann cell differentiation	1.49E−02	**EGR2**
GO:0044843	Cell cycle G1/S phase transition	1.54E−02	**CDKN1A**;*GSPT1*
GO:0060441	Epithelial tube branching involved in lung morphogenesis	1.79E−02	*NKX2-1*
GO:0032020	ISG15-protein conjugation	1.79E−02	*ISG15*
GO:0060373	Regulation of ventricular cardiac muscle cell membrane depolarization	1.79E−02	*SCN3B*
GO:0044058	Regulation of digestive system process	1.79E−02	**SGK1**
GO:0060371	Regulation of atrial cardiac muscle cell membrane depolarization	2.08E−02	*SCN3B*
GO:0086015	SA node cell action potential	2.08E−02	*SCN3B*

GO ID	Molecular function description	p-value	DEGs
GO:0017080	Sodium channel regulator activity	5.51E−03	*SCN3B;* **SGK1**
GO:0086006	Voltage-gated sodium channel activity involved in cardiac muscle cell action potential	1.49E−02	*SCN3B*
GO:0019871	Sodium channel inhibitor activity	2.38E−02	*SCN3B*
GO:0004861	Cyclin-dependent protein serine/threonine kinase inhibitor activity	2.96E−02	**CDKN1A**
GO:0061665	SUMO ligase activity	2.96E−02	**EGR2**
GO:0017081	Chloride channel regulator activity	4.41E−02	**SGK1**
GO:0004129	Cytochrome-c oxidase activity	4.41E−02	**COX4I2**
GO:0051371	Muscle alpha-actinin binding	4.41E−02	*PDLIM1*
GO:0031625	Ubiquitin protein ligase binding	4.53E−02	**CDKN1A;EGR2;** *ISG15*
GO:0010314	Phosphatidylinositol-5-phosphate binding	4.70E−02	**PLEKHF1**

Using EnrichR, differentially regulated gene lists were evaluated for significant enrichment against the following gene set libraries: GO Biological Process, GO Molecular Function (all from http://www.geneontology.org). The top 10 enriched terms were selected and ranked based upon the combined score that was calculated by the EnrichR platform following Z-score permutation background correction on the Fisher exact test p-value. Results were filtered for p < 0.05. Upregulated genes in bold and downregulated genes in italics.

**Table 2:** Gene ontology of differentially expressed genes in the PVN of emulsifier treated mice.

Gene ontology functional enrichment analysis			
PVN—CMC vs water			
GO ID	Biological process description	p-value	DEGs
GO:0090209	Negative regulation of triglyceride metabolic process	9.88E−05	**APOC3;SIK1**
GO:0045601	Regulation of endothelial cell differentiation	2.36E−04	**ATOH8;APOLD1**
GO:0048660	Regulation of smooth muscle cell proliferation	2.81E−04	*EDN1*;**NR4A3;TRIB1**
GO:0010613	Positive regulation of cardiac muscle hypertrophy	9.88E−04	*EDN1*;**NR4A3**
GO:0046887	Positive regulation of hormone secretion	1.22E−03	*EDN1;GRP*
GO:0051004	Regulation of lipoprotein lipase activity	1.35E−03	*ANGPTL8*;**APOC3**
GO:0051055	Negative regulation of lipid biosynthetic process	1.48E−03	**APOC3;SIK1**
GO:0010611	Regulation of cardiac muscle hypertrophy	1.76E−03	*EDN1*;**NR4A3**
GO:0055090	Acylglycerol homeostasis	1.91E−03	*ANGPTL8*;**APOC3**
GO:0070328	Triglyceride homeostasis	2.94E−03	*ANGPTL8*;**APOC3**

GO ID	Molecular function description	p-value	DEGs
GO:0016615	Malate dehydrogenase activity	1.55E−02	*ME3*
GO:0005179	Hormone activity	1.76E−02	*EDN1;GRP*
GO:0031434	Mitogen-activated protein kinase kinase binding	2.06E−02	**TRIB1**
GO:0035259	Glucocorticoid receptor binding	2.32E−02	**NR4A3**
GO:0008140	cAMP response element binding protein binding	2.32E−02	**SIK1**
GO:0035497	cAMP response element binding	2.57E−02	**NR4A3**
GO:0055102	Lipase inhibitor activity	2.57E−02	**APOC3**

PVN—P80 vs water			
GO ID	Biological process description	p-value	DEGs
GO:0007616	Long-term memory	2.34E−03	**NPAS4;SGK1**
GO:0046329	Negative regulation of JNK cascade	6.97E−03	**PER1;** *HIPK3*
GO:0048660	Regulation of smooth muscle cell proliferation	1.49E−02	**NR4A3;TRIB1**
GO:0007614	Short-term memory	1.89E−02	*NPAS4*
GO:1903208	Negative regulation of hydrogen peroxide-induced neuron death	1.89E−02	**NR4A3**
GO:2000253	Positive regulation of feeding behavior	1.89E−02	**NR4A3**
GO:1903749	Positive regulation of establishment of protein localization to mitochondrion	1.92E−02	**PMAIP1;RHOU**
GO:0015820	Leucine transport	2.26E−02	**SLC6A17**
GO:0061469	Regulation of type B pancreatic cell proliferation	2.26E−02	**NR4A3**
GO:2000323	Negative regulation of glucocorticoid receptor signaling pathway	2.26E−02	**PER1**

GO ID	Molecular function description	p-value	DEGs
GO:0051998	Protein carboxyl O-methyltransferase activity	2.26E−02	**PCMTD2**
GO:0031995	Insulin-like growth factor II binding	2.63E−02	*IGFBP2*
GO:0031434	Mitogen-activated protein kinase kinase binding	3.00E−02	**TRIB1**
GO:0035259	Glucocorticoid receptor binding	3.37E−02	**NR4A3**
GO:0035497	cAMP response element binding	3.74E−02	**NR4A3**
GO:0046975	Histone methyltransferase activity (H3-K36 specific)	4.10E−02	*PRDM9*
GO:0070679	Inositol 1,4,5 trisphosphate binding	4.10E−02	**CYTH2**
GO:0070513	Death domain binding	4.10E−02	*DAPL1*
GO:0031994	Insulin-like growth factor I binding	4.83E-02	*IGFBP2*

Using EnrichR, differentially regulated gene lists were evaluated for significant enrichment against the following gene set libraries: GO Biological Process, GO Molecular Function (all from http://www.geneontology.org). The top 10 enriched terms were selected and ranked based upon the combined score that was calculated by the EnrichR platform following Z-score permutation background correction on the Fisher exact test p-value. Results were filtered for p < 0.05. Upregulated genes in bold and downregulated genes in italics.

### Enriched pathways

**Figure 6:**
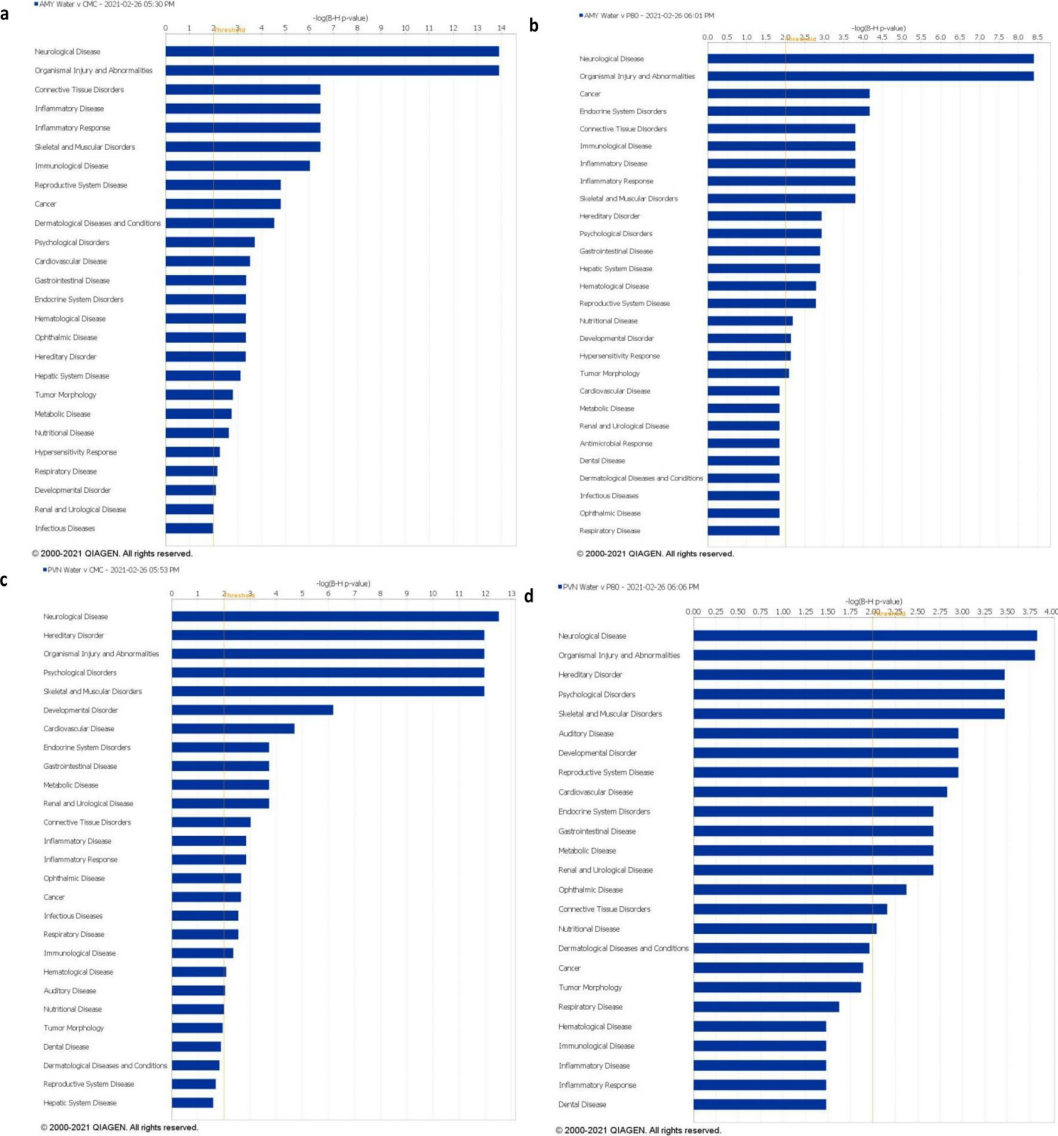
Top enriched IPA disease pathways. Top diseases in the amygdala after CMC treatment (**a**) or P80 treatment (**b**) and PVN after CMC treatment (**c**) or P80 treatment (**d**). Threshold indicates minimum significance level [–log (p-value) from Fisher’s exact test].

To gain a mechanistic understanding of the altered gene expression observed during emulsifier consumption, we next performed bioinformatic analysis using INGENUITY^®^ Pathway Analysis (IPA) to examine the biological relevance of altered gene pathways using a systems biology approach. Genes imported into IPA had a stringency filter set for statistically significant genes (fold-change > 1, adj *p*-value < 0.05) within the amygdala and PVN of emulsifier vs water controls. The top 5 canonical pathways (CPs) are presented in Table 3. These analyses revealed sets of genes within the amygdala and PVN tissue that were highly enriched in primarily immune-related canonical pathways. Interestingly, though both CMC and P80 conditions resulted in enriched immune-related pathways within the amygdala and PVN, neither condition had a common pathway shared. Among CMC-treated animals, the top dysregulated canonical pathways in the amygdala were MIF regulation of innate immunity (p = 9.47E−05), corticotropin-releasing hormone (CRH) signaling (p = 2.45E−04), Coronavirus pathogenesis pathway (p = 2.79E−04), CD40 signaling (p = 3.48E−04), and ILK signaling (p = 6.81E−04). Within the PVN, the top canonical pathways included opioid signaling pathway (p = 4.14E−06), G-protein coupled receptor signaling (p = 8.10E−05), CRH signaling (p = 9.13E−05), CREB signaling in neurons (p = 1.95E−04), and CDK5 signaling (p = 2.19E−04). Figure 5 depicts the IPA predicted molecular interactions between proteins of DEGs within the CRH pathway. Functionally, glucocorticoid synthesis may be Nur77/Nr4a1 dependent and pro-inflammatory prostaglandin synthesis may be COX2 dependent within the canonical CRH pathway. Among P80-treated animals, the top dysregulated canonical pathways in the amygdala were IL-17A signaling in gastric cells (p = 1.08E−03), T-cell receptor signaling (p = 1.39E−03), CD28 signaling in T-helper cells (p = 1.67E−03), TNFR2 signaling (p = 1.84E−03), and IL-17A signaling in fibroblasts (p = 2.22E−03). Within the PVN, the top canonical pathways included role of JAK2 in hormone-like cytokine signaling (p = 6.22E−04), opioid signaling pathway (p = 1.52E−03), role of Wnt/GSK-3 signaling in the pathogenesis of Influenza (p = 4.63E−03), human embryonic stem cell pluripotency (p = 5.02E−03), and role of JAK1 and JAK3 in cytokine signaling (p = 5.49E−03). In addition, analysis of significant disease pathways in IPA showed considerable overlap between emulsifier conditions but also showed regional differences that likely reflect the region-specific response to emulsifier treatment (Fig. 6). Altogether, these results demonstrate that CMC and P80 consumption induces limited but physiologically relevant alterations in gene expression in brain regions controlling physiological and behavioral concomitants of stress and anxiety.

**Table 3:** Top five IPA enriched canonical pathways of differentially expressed genes in the amygdala and PVN of emulsifier treated mice.

Top canonical pathways		
Name	p-value	Overlap
**Amygdala—CMC vs water**		
MIF regulation of innate immunity	9.47E−05	7.1% 3/42
Corticotropin releasing hormone signaling	2.45E−04	2.8% 4/145
Coronavirus pathogenesis pathway	2.79E−04	2.7% 4/150
CD40 signaling	3.48E−04	4.6% 3/65
ILK signaling	6.81E−04	2.1% 4/190
**Amygdala—P80 vs water**		
IL-17A signaling in gastric cells	1.08E−03	8.7% 2/23
T cell receptor signaling	1.39E−03	2.9% 3/104
CD28 signaling in T helper cells	1.67E−03	2.7% 3/111
TNFR2 signaling	1.84E−03	6.7% 2/30
IL-17A signaling in fibroblasts	2.22E−03	6.1% 2/33
**PVN—CMC vs water**		
Opioid signaling pathway	4.14E−06	3.8% 9/236
G-Protein coupled receptor signaling	8.10E−05	3.0% 8/267
Corticotropin releasing hormone signaling	9.13E−05	4.3% 6/139
CREB signaling in neurons	1.95E−04	1.9% 11/570
CDK5 signaling	2.19E−04	4.8% 5/104
**PVN—P80 vs water**		
Role of JAK2 in hormone-like cytokine signaling	6.22E−04	9.4% 3/32
Opioid signaling pathway	1.52E−03	2.5% 6/236
Role of Wnt/GSK-3 signaling in the pathogenesis of influenza	4.63E−03	4.7% 3/64
Human embryonic stem cell pluripotency	5.02E−03	3.1% 4/131
Role of JAK1 and JAK3 in c cytokine signaling	5.49E-03	4.4% 3/68

Emulsifier-treatment is primarily associated with inflammation and other immune-related canonical pathways. The top categories were ranked in accordance with their P-value of overlap. Overlap refers to the number of molecules from the dataset that map to the pathway listed divided by the total number of molecules that define the canonical pathway from within the IPA knowledgebase.

### Validation of RNA-Seq results by quantitative real-time PCR (qRT-PCR)

Six DEGs were selected due to their involvement in stress responding and immune function according to previous studies. SGK1, NR4A3, PRRC2A, FOSB, PTGS2, and EGR2 were selected to validate RNA-Seq results by qRT-PCR (Supplemental Table 1). Expression levels calculated via RNA-Seq were significantly positively correlated to expression levels determined via qRT-PCR (Supplemental Fig. 1; R2 = 0.8815, p < 0.0001). For all genes tested, qRT-PCR data strongly correlate with RNA-Seq data except for PTGS2 expression levels within the PVN. Expression levels of PTGS2 measured by qRT-PCR were higher than indicated by RNA-Seq expression. Overall, the correlation observed between qRT-PCR and RNA-seq further strengthens the emulsifier-induced alteration in gene expression described above, validating the RNA-seq approach on punched brain regions to investigate the impact of altered host/microbiota relationship on the central nervous system.

### Consumption of dietary emulsifiers does not result in dehydration

One potential confounding factor that could drive gene expression changes in the current study is changes in hydration among groups if the emulsifiers altered water intake or absorption^22^. To eliminate the possibility that gene changes were secondary to changes in hydration rather than to the emulsifier treatment, itself, various hydration measures were compared between emulsifier-treated animals and water controls. Measures included liquid intake, plasma osmolarity, and a hydration ratio obtained by echo magnetic resonance imaging (eMRI). There were no significant differences in liquid intake between groups (CMC vs water, p = 0.86 and P80 vs water, p = 0.19) over 3 days at the end of the treatment phase of the study (Supplemental Fig. 2a). Additionally, neither the hydration ratio of CMC (p = 0.47) nor P80 (p = 0.12) differed significantly from water-treated controls (Supplemental Fig. 2b). There were no significant differences in plasma osmolarity, a measure of hydration status, between groups (CMC vs water, p = 0.78 and P80 vs water, p = 0.52) (Supplemental Fig. 2c). Altogether, all three hydration measures indicate that emulsifier treatment does not result in changes in liquid intake, dehydration, or changes in body composition, thus this is unlikely to be a confounding factor driving differential gene expression seen in emulsifier conditions versus control.

## Discussion

The present study tested the hypothesis that emulsifier intake, at doses that are directly relevant to those ingested by humans, can influence gene expression in brain regions that are known to be important in the generation of behavioral and neuroendocrine responses to stress-provoking stimuli. The current findings illustrate the novel finding that emulsifier consumption induces genetic alternations within the amygdala and PVN that could be associated with the previously reported anxiety-like phenotype^7^. In particular, it appears that emulsifier treatment increased expression of IEGs such as NR4A2, NR4A3, EGR2, JUNB, FOSB, and FOSL2 in the amygdala and PVN, suggesting that both CMC and P80 may increase neuronal activity in brain regions that modulate stress responding. Because no additional procedures were experienced by the animals before euthanasia, the increase in IEGs suggests that the emulsifiers may have increased sensitivity to the mild stress of transport and handling that occurred just before tissue collection.

Increased neural activity in the amygdala and PVN is associated with an anxiety-like phenotype in rodents and may thus be brain regions within which emulsifiers act to increase anxiety-like behavior^23^. Further, differential expression of multiple HPA axis regulatory and responsive genes in emulsifier-treated animals indicates possible dysregulation of the HPA axis. Analysis of gene ontology shows emulsifier-induced alterations in the function of “glucocorticoid receptor binding” in both CMC and P80 conditions. NR4A2, NR4A3, and SGK1 are known to code for proteins that regulate glucocorticoid receptor function, and all three of these showed increased differential expression in emulsifier-treated animals. NR4A2 (NURR1) and NR4A3 (NOR1) are immediate early genes induced by growth factors, inflammatory signals, and glucocorticoids and are known to regulate the HPA axis at the hypothalamic, pituitary, and adrenal level^24,25^. In addition, we found emulsifier-treated animals had decreased expression of PCLO and increased expression of SGK1, which are known biomarkers of clinical depression, a disorder often characterized by HPA axis dysregulation. More specifically, decreased expression of PLCO is associated with increased activity in the amygdala of depressed patients^26^. Increased expression of SGK1, a glucocorticoid receptor (GR)-inducible gene, is found in the hypothalamus and the serum of patients with depression^20^. SGK1 is known to prolong GR activation^27^, which makes this gene a prime, potential molecular mechanism by which emulsifiers may sensitize the stress response.

Neuroinflammation is another possible mechanism by which emulsifier consumption may alter behavior. It is previously established that emulsifier consumption causes low-grade inflammation in the periphery^6,28^. The present study is the first to provide evidence that emulsifiers may cause immune activity centrally, as well. The top enriched canonical pathways in emulsifier-treated animals were largely comprised of altered immune pathways in both the amygdala and PVN. These potential changes in central immune activation are highly relevant, as increased neuroinflammation is proposed to be a driving factor in the expression of anxiety and depression-like behavior^29^. Indeed, recent studies have demonstrated that decreasing innate immune system activity ameliorates social defeat-induced social anxiety and depression in mice^29^. One immune DEG of high interest that increased in the amygdala as a result of emulsifier consumption was PTGS2 (COX2), a gene responsible for coding the enzyme that converts arachidonic acid to pro-inflammatory prostaglandins. Other studies show that increased COX2 expression in the brain increases susceptibility to chronic mild unpredictable stress in rats, and COX2 inhibition decreases anxiety-like behavior in mice and decreases glutamatergic activity in the amygdala^30,31^. Taken together, Cox-2 may be another prime mechanistic candidate driving emulsifier-induced stress sensitivity. Further connecting neuro- inflammation to stress sensitivity, the top canonical pathway identified in the amygdala of CMC-treated mice was “MIF Regulation of Innate Immunity”. Macrophage migration inhibitory factor (MIF) is released from immune cells in response to infectious stimuli and is also considered an endocrine hormone capable of participating in HPA axis regulation^32^. Moreover, MIF is highly expressed in the brain and plays a key role in anxiety- and depression-like behaviors^33,34^.

It is important to note that both blood and vasculature were likely present in our brain samples. Several genes expressed in vasculature endothelial cells were found to be altered in the PVN and amygdala of emulsifier-treated animals and are known to play a role in blood–brain barrier function. Decreased expression of PCDHGA5, the gene that codes for a cadherin-like adhesion protein, was found in the PVN of CMC-treated mice. This gene plays a critical role in the barrier-stabilization properties of the blood–brain barrier (BBB)^35^. Decreased expression of this gene may indicate compromised vasculature within the brain. In addition, increased expression of ARNO (cytohesion-2) was found in the PVN of P80-treated mice. This gene plays a critical role in the permeability of the BBB. Increased expression of ARNO is associated with increased vessel permeability^36^. Lastly, increased expression of SPARC was found in the amygdala of CMC-treated mice. Pro-inflammatory molecules are known to increase SPARC expression in endothelial cells. SPARC alters BBB properties through increased paracellular permeability and decreased transendothelial electrical resistance (TEER)^37^. The presence of these genes is of particular interest as both neuroinflammation and BBB dysfunction are implicated in the neurobiology of stress-related disorders, namely, major depressive disorder^38^.

A limitation to this study is the exclusion of females from our dataset. Due to the high per sample cost of RNA-seq, only males were included in this study. Diet-induced inflammation may have sex-specific effects on gene expression in areas of the brain critical for stress responding, such as the amygdala and PVN^39^. The exclusion of females from this study is a clear limitation, as sex differences in the prevalence of both inflammatory disease and stress-related disorders exist in the clinical population^40,41^. A critical future direction will be to investigate sex differences in emulsifier-induced gene expression in brain regions critical for the modulation of the stress response^7,42^. Another consideration is the fact that the 1 mm tissue punches of PVN and amygdala clearly captured several distinct subregions. Therefore, the DEGs captured here are summative within these heterogeneous regions and therefore could have missed important changes within smaller subareas within each punch. Future studies could use single-cell RNA-Seq to examine DEGs in identified cell groups.

In conclusion, these data are the first to show that chronic emulsifier ingestion, known to be associated with microbiota alterations and low-grade inflammation, induced altered gene expression in key areas of the brain that control stress-related behavior. The transcripts and pathways highlighted here provide a more concrete understanding of the molecular mechanisms by which emulsifier consumption may affect HPA-axis functioning and stress-associated behaviors. Future experiments will test the extent to which emulsifiers sensitize behavioral and neuroendocrine stress responding, specifically assessing the role of the discussed DEGs in driving these phenotypes. Ultimately, the data obtained from this project highlight potential molecular neural mechanisms by which food additives, like emulsifiers, may affect inflammatory processes and behavior.

## Methods

### Animals

Adult male C57BL/6J mice, 8 weeks of age (The Jackson Laboratory, Bar Harbor, ME), were group-housed (5 mice per cage) in ventilated OptiMouse plastic cages with AlphaDri bedding. Mice were maintained on a 12:12 h light/dark cycle, and food and water were supplied ad libitum. All procedures were approved by and performed in accordance with the guidelines of the Institutional Animal Care and Use Committee (IACUC) at Georgia State University and with national regulations and policies. This study was performed in accordance with Animals in Research: Reporting In Vivo Experiments (ARRIVE) guidelines.

### Procedure

Mice (n = 5/group) received drinking water containing either 1% sodium carboxymethylcellulose (CMC; Sigma, St. Louis, MO), 1% polysorbate-80 (P80; Sigma), or water without an emulsifier, a control group. Our previous work has shown that similar inflammatory and metabolic changes occur following emulsifiers administered in either food or water^6^. Body weights were measured throughout the duration of the study. After 12 weeks of respective emulsifier or control treatment, animals were removed from their home cage, immediately anesthetized with isoflurane, and then decapitated. Brains were rapidly extracted, flash-frozen in ice-cold isopentane, and then stored at – 80 °C until sectioning. Fat-pads, colons, and spleens were also collected for analysis of weight or length (colon).

### RNA extraction

Brains were sectioned coronally at 300 μm on a cryostat and bilateral 1 mm tissue punches were taken of the central/basolateral amygdala and the PVN. For RNA-seq and real-time, quantitative polymerase chain reaction (RT-qPCR), total RNA was extracted from tissue punches using a Trizol extraction method as described earlier^6^. In brief, brain tissue was homogenized in Trizol, followed by the addition of chloroform, and then centrifuged to separate the RNA aqueous layer from other cell contents. RNA was then alcohol-precipitated and resuspended in 30 μl nuclease-free water. RNA concentration and purity were measured using a nanodrop spectrophotometer, and the two samples from each group with the highest concentration and purity were sent for Illumina sequencing as described below.

### mRNA sequencing library preparation

Total RNA extracted from the bilateral amygdala and PVN punches of animals treated with either CMC, P80, or emulsifier-free drinking water (N = 2/group) were used to create mRNA sequencing libraries. Our initial study used a small number of samples taken from individual animals to maximize the number of group comparisons, while still allowing for the statistical identification of possible differentially expressed genes that could be subsequently validated with quantitative PCR using a larger group of animals^43,44^. The mRNA sequencing library was constructed using the TruSeq RNA Sample Preparation kit (Illumina, San Diego, CA) following the manufacturer’s instructions. In brief, PolyA-containing mRNAs were purified using oligo-dT attached magnetic beads followed by RNA fragmentation. cDNA was synthesized from RNA primed with random hexamers using reverse transcriptase. 3′ overhangs and 5′ overhangs were repaired using End Repair Mix. After adenylation of the 3′ end, indexing adaptors are added to cDNA in preparation to be hybridized onto a flow cell. PCR was performed with a PCR Primer Cocktail that selectively amplifies DNA fragments with adaptors.

### Sequencing

Each sample was uniquely indexed (barcoded) to allow for the pooling of all samples in a single sequencing run. A 75 cycle sequencing run was performed on the Illumina NextSeq 500 at the Genomics Core at Cornell University (Ithaca, NY). Data were processed using the standard Illumina processing pipeline to segregate each multiplexed samples’ reads. The resulting data files were in fastq format and included at least 20 million reads per sample.

### Analysis pipeline

Various programs were used to assess sequence quality, determine differential gene expression, and perform ontological and pathway analysis. The FastQC program (https://www.bioinformatics.babraham.ac.uk/projects/fastqc/) was used to assess the quality of reads and to provide various statistics such as total reads, total sequences, GC content, and sequence duplication levels. GC content for each sample fit the theoretical distribution, thus indicating no contamination or systemic bias within the samples. FASTQ quality filter (http://hannonlab.cshl.edu/fastx_toolkit/commandline.html#fastq_quality_filter_usage) was used to filter low-quality sequences (base quality score less than 30) leaving only sequences with high-quality scores. High-quality reads were then aligned to mm10 mus musculus reference genome using Bowtie2 and Tophat2 to identify gene sequences^45^. Gene expression levels were measured using Cufflinks and differential expression was measured using Cuffdiff^46^. Fragments Per Kilobase of transcript per Million mapped reads (FPKM) expression scores were obtained for each gene. Log2fold differential expression scores for each gene are calculated by dividing the average FPKM score for each experimental group by the average FPKM score for the water control group and converting the fold change to a logarithmic 2 scale after which a p-value was calculated^47^. P-score values were FDR adjusted (q-value) using the Benjamini–Hochberg correction^48^. Only genes that had an adjusted p-score value (q-value) < 0.05 and at least a log2fold score of 1 and − 1, which are widely accepted cutoffs, were used for subsequent analysis^49^. Lastly, EnrichR and Ingenuity Pathway Analysis (QIAGEN Inc., https://digitalinsights.qiagen.com/IPA) were used to perform gene set enrichment and to analyze DEGs to predict significant canonical pathways and diseases. Overlap with canonical pathways were based on well-established metabolic and signaling pathways that are within the INGENUITY knowledgebase, and enrichment for diseases were based on the annotations for diseases and disorders in the IPA curated knowledgebase. The Fisher's Exact Test was used to calculate the statistical significance of the overlap of DEG. R was used to generate expression heat maps using the heatmap.2 function from the ggplot2 library and volcano plots were generated using in-house scripts.

### RT-qPCR validation

Six genes of interest that were observed to be differentially expressed in the RNA-Seq dataset were selected for further RT-qPCR validation. Animals (n = 5/group) were treated as described in Experiment 1 (RNA-Seq) and then gene expression was measured in the amygdala and PVN. In brief, RNA from tissue punches of the amygdala and PVN was synthesized into cDNA using a SuperScript IV First Stand Synthesis System to manufacture’s specifications. Primers for genes of interest were designed using the NCBI primer design tool and only sequences that were exclusively specific to the gene of interest were used. Primer efficiency and specificity were verified; primer sequences are listed in Supplementary Table 1. cDNA was amplified using primers and Quantifast SYBR green PCR reaction mix via real-time PCR to allow for quantification of total gene expression. PCR cycle conditions were 95 °C for 5 min then 40 amplification cycles of 95 °C for 10 s to denature and 60 °C for 30 s to anneal and elongate. A melting curve was run for each primer to verify the formation of only one product. Data were normalized to the well-validated housekeeping gene, YWHAZ using the ΔΔct method because previous research has shown that YWHAZ expression remains stable under inflammatory conditions^50^, and we verified that it did not vary by condition in this study. RT-qPCR relative gene expression within each brain region was compared between emulsifier and water control groups.

### Hydration measures

#### Liquid intake

To verify that the emulsifier treatment did not alter the volume of liquid ingested, liquid intake was measured in an additional cohort of animals (N = 10/group) that were also treated as in Experiment 1 (RNA-Seq). Thus, after 12-weeks of emulsifier or control treatment, animals were singly housed to determine the volume ingested by each individual over a 72 h period. Mice were given a 50 ml sipper tube filled with respective emulsifier or water. Volume within the sipper bottle was logged daily for 3 consecutive days to determine the total ml of fluid ingested.

#### Echo magnetic resonance imaging (eMRI)

In addition to the liquid intake measures, the body composition of each subject was measured using an EchoMRI 1100 (EchoMRI LLC, Houston, TX, USA) to determine whether emulsifier intake altered body composition or hydration compared to that observed in mice drinking only water with no emulsifier added. Briefly, animals were weighed and inserted into the eMRI restrainer tube. The tube was then inserted into the eMRI machine and body composition was measured. This procedure is non-invasive and does not require the use of anesthesia. Tubes were disinfected with 70% ethanol between testing each mouse. Parameters were measured in triplicate with each measure lasting approximately 30 s. Measures collected included body mass, total body fat, lean body mass, free water, and total body water content. A hydration ratio was obtained by using the following equation: (total water – free water/ lean mass).

#### Plasma osmolarity

Immediately after the completion of eMRI measures, animals were sacrificed by decapitation and trunk blood was collected in heparinized tubes. Blood was spun down immediately in a refrigerated microcentrifuge at 1500×*g* for 10 min. The plasma layer was isolated and plasma osmolality was quantitatively determined using the Advanced Instruments Model 3300 Micro*-*Osmometer (Advanced Instruments, Norwood, MA, USA) according to the manufacturer’s instructions.

## Supplementary Information

Supplementary Information.

## Data Availability

Raw sequence and processed RNA-Seq data generated in this research have been deposited in the NCBI's Gene Expression Omnibus under accession number GSE194260 and are publicly accessible at https://www.ncbi.nlm.nih.gov/geo/query/acc.cgi?acc=GSE194260.
