# Supplementary material for: Dietary emulsifier consumption alters gene expression in the amygdala and paraventricular nucleus of the hypothalamus in mice
Source: Sci Rep. 2022 Jun 1;12:9146. doi: 10.1038/s41598-022-13021-7 (PMC9159048; doi:10.1038/s41598-022-13021-7)
Supplement: Supplementary file 1 — Supplementary Information. [file 41598_2022_13021_MOESM1_ESM.docx]

**Title:**Dietary emulsifier consumption alters gene expression in the amygdala and paraventricular nucleus of the hypothalamus in mice

**Authors:**
Amanda R. Arnold, Benoit Chassaing, Bradley D. Pearce, Kim L. Huhman

**Supplemental Tables and Figures:**

| qRT-PCR Primers | | |
| --- | --- | --- |
| **Gene** | **Forward-sequence** | **Reverse-sequence** |
| Sgk1 | TCCTGAGGTCCTCCATAAGCA | GTGCCTTGCCGAGTTTGTAAT |
| Nr4a3 | TTCTGACGGCCTCCATTGAC | CTCCCCAAATCCTCGAAGGC |
| Prrc2a | GGACTCTGCCGGGGTTAAT | AGAAGACCTCAGGGTACGGA |
| Fosb | GCCAGGAACCAGCTACTCAA | CTTGTTCCGCTCTCTGCGAA |
| Ptgs2 | CTGACCCCCAAGGCTCAAAT | TCTGCTCTGGTCAATGGAGG |
| Egr2 | GCCGTAGACAAAATCCCAGT | AGCTACTCGGATACGGGAGA |
| Ywhaz | GATCCCCAATGCTTCGCAAC | CCAGTCTGATGGGGTGTGTC |

**Supplemental Table S1**: List of primer sequences used for qRT-PCR.


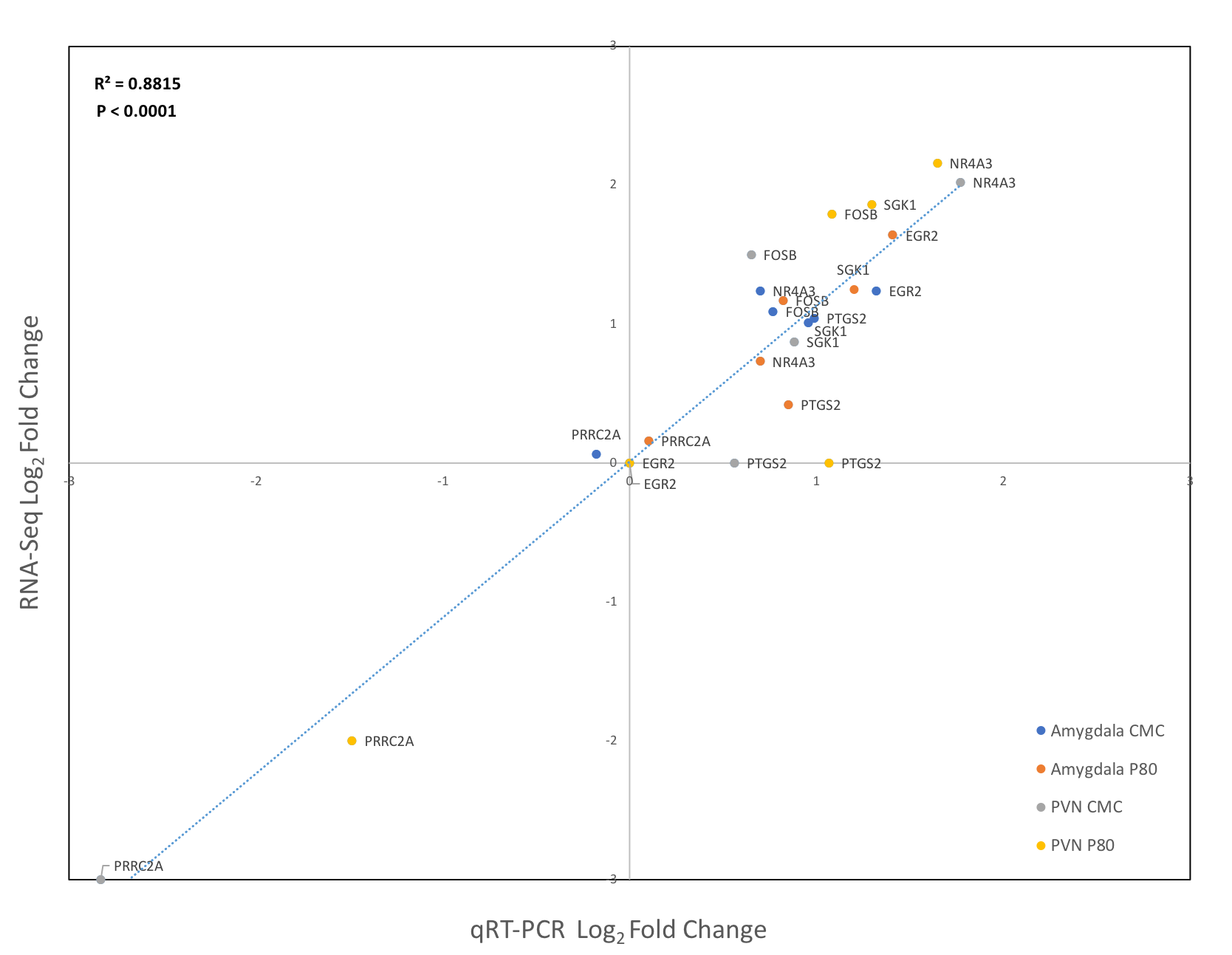


**Supplemental Figure 1**: Validation of RNA-Sequencing results by quantitative RT-PCR. Log2 fold change correlation between RNA-Seq and quantitative real-time PCR (qRT-PCR) for six differentially expressed genes (SGK1, NR4A3, PRRC2A, FOSB, PTGS2, EGR2; n=5) within Amygdala CMC, Amygdala P80, PVN CMC, and PVN P80 conditions. Correlation between RNA-Seq and qRT-PCR gene expression (R2=0.8815, p<0.0001).

**Supplemental Figure 2:** Hydration measures. There were no differences among groups in liquid intake (a), hydration ratio as measured by eMRI (b), or plasma osmolarity (c). N=10 in each group.
